# The Psycho-Social Impact of Dental Emergencies in COVID-19 Patients: A Cross-Sectional Case–Control Study

**DOI:** 10.3390/diseases14030087

**Published:** 2026-02-26

**Authors:** Marius Moroianu, Ramona-Oana Roșca, Laura-Carmen Cristescu-Budala, Valeriu Ardeleanu, Iulian Bounegru, Mădălina Nicoleta Matei

**Affiliations:** 1 Department of Dental Medicine, Faculty of Medicine and Pharmacy, “Dunărea de Jos” University of Galați, 800201 Galați, Romania; moroianu.g.marius@gmail.com (M.M.); madalina.matei@ugal.ro (M.N.M.); 2 Department of Pharmaceutical Sciences, Faculty of Medicine and Pharmacy, “Dunărea de Jos” University of Galați, 47 Domnească Street, 800008 Galați, Romania; ramona.rosca@ugal.ro; 3 Department of Medical Disciplines, Faculty of Medicine and Pharmacy, “Dunărea de Jos” University of Galați, 800201 Galați, Romania; laura.cristescubudala@gmail.com; 4 Faculty of Kinesiotherapy, “Dunărea de Jos” University of Galați, 800008 Galați, Romania; 5 Competences Centre: Interfaces-Tribocorrosion-Electrochemical Systems, “Dunărea de Jos” University of Galați, 800008 Galați, Romania

**Keywords:** dental emergency, COVID-19, quality of life, QOLI, ordinal logistic regression, effect size

## Abstract

**Background:** The COVID-19 pandemic severely restricted access to routine dental care, resulting in delayed treatment and increased presentation of dental emergencies. When combined with SARS-CoV-2 infection, these conditions may significantly impair psycho-social well-being and quality of life (QoL). This study assessed the impact of dental emergencies on QoL in patients with COVID-19. **Methods:** A cross-sectional case–control study was conducted between January 2022 and April 2024, including 240 adult patients with confirmed COVID-19. The case group comprised 60 patients presenting with dental emergencies, while the control group included 180 COVID-19 patients without emergency dental needs. Quality of life was evaluated using the 32-item Quality-of-Life Inventory (QOLI), yielding a continuous global score (SBQ) and an ordinal quality-of-life category (CGV). Group comparisons were performed using Welch’s *t*-test and logistic regression, with effect sizes and 95% confidence intervals reported. Multivariable analyses were adjusted for age and sex. **Results:** Patients with dental emergencies reported markedly poorer global QoL compared to controls (mean SBQ difference = −2.04 points; Cohen’s d = −1.50; *p* < 0.001). The presence of a dental emergency was strongly associated with severe QoL impairment, with emergency patients showing substantially higher odds of unfavorable CGV categories (adjusted OR ≈ 20.4; 95% CI: 8.6–48.5; *p* < 0.001). These associations remained robust after adjustment for demographic covariates. **Conclusions:** Dental emergencies in patients with COVID-19 are associated with a profound deterioration in quality of life. Ensuring timely access to emergency dental services during public health crises may substantially reduce psycho-social burden and improve patient-centered outcomes.

## 1. Introduction

The coronavirus disease 2019 (COVID-19) pandemic profoundly disrupted dental care delivery worldwide, producing a rapid shift from routine and elective procedures to the provision of urgent and emergency services only. Reports from Europe and Asia consistently documented sharp reductions in overall patient volume, accompanied by a relative increase in painful and infection-related conditions that could not be deferred [1,2,3]. In many health systems, professional bodies explicitly recommend limiting care to dental emergencies during periods of high community transmission. While justified from an infection-control perspective, these policies inevitably generated treatment delays, disease progression, and increased symptom burden [4]. Subsequent service evaluations confirmed that emergency dental care during lockdown was delivered to patients with more advanced pathology, such as severe pulpitis, abscesses, or trauma, and that invasive procedures (e.g., extractions or incision and drainage) became proportionally more frequent than conservative treatments [2,4,5]. Beyond somatic morbidity, SARS-CoV-2 infection itself has been associated with a high prevalence of psychopathological symptoms, including anxiety, depression, fear, and insomnia, which likely amplified the perceived impact of acute dental problems on overall quality of life (QoL) during the pandemic [6].

Evidence from pediatric and adolescent populations illustrates the broad psychosocial impact of the COVID-19 pandemic on healthcare experiences. Multiple studies have shown that children and adolescents perceived medical settings during the pandemic as more threatening and unpredictable, reporting increased levels of fear, anxiety, and healthcare avoidance behaviors. These responses were largely driven by infection-control measures, changes in care routines, limited communication, and restricted caregiver presence during medical visits [7,8,9]. Systematic reviews and multicenter studies further indicate that pandemic-related disruptions altered emotional appraisal of healthcare environments, with lasting effects on trust, perceived safety, and engagement with health services among younger patients [10,11]. Collectively, these findings suggest that pandemic-related stressors reshaped patients’ emotional responses to healthcare encounters across age groups, providing relevant context for understanding similar psychosocial mechanisms in adults presenting with acute dental emergencies during COVID-19.

During the study period, Romania experienced a substantial burden from the COVID-19 pandemic, with cumulative confirmed cases and fatalities among the highest in Eastern Europe. According to global surveillance data, Romania reported over 3.5 million confirmed cases of COVID-19 and approximately 68,900 related deaths by early 2024, reflecting widespread community transmission and multiple waves of infection over 2020–2023. These data underscore the persistent public health impact of SARS-CoV-2 in the country.

Romania experienced a substantial burden from the COVID-19 pandemic, with cumulative confirmed cases and fatalities among the highest in Eastern Europe. By early 2024, over 3.5 million confirmed cases and approximately 68,900 related deaths had been reported, reflecting widespread community transmission and multiple infection waves between 2020 and 2023 [12]. The national healthcare system underwent repeated reorganizations during this period, including suspending routine services and prioritizing urgent care, in line with evolving guidance from the European Centre for Disease Prevention and Control (ECDC) [12,13,14]. Within this context, elevated levels of anxiety and fear concerning healthcare—including dental care—were widely documented. These were driven by fear of infection, uncertainty regarding clinic safety, and concerns about aerosol transmission during dental procedures. Dental anxiety, already a well-recognized phenomenon before the pandemic, is associated with fear of pain, previous negative experiences, and anticipatory stress related to dental visits. A Romanian study reported that anxiety related to dental appointments was prevalent even in routine care settings and was significantly associated with avoidance behaviors and delayed presentation to dental services. During COVID-19, cross-sectional surveys showed that many dental patients reported moderate to high levels of fear related to attending dental appointments, despite implemented infection-control measures, and that concerns about COVID-19 substantially influenced care-seeking behavior [15,16].

Dental emergencies are uniquely detrimental to daily functioning because they combine acute nociceptive pain, eating and speaking difficulties, sleep disturbance, and visible orofacial changes. Long before the pandemic, oral-health-related quality-of-life (OHRQoL) research demonstrated that dental pain and infection produce some of the largest decrements on both generic and oral-specific QoL instruments, often comparable to those observed in other chronic conditions [15,16]. The pandemic introduced additional stressors—quarantine, fear of contagion, income loss, and social isolation—that further magnified the perceived impact of oral symptoms. Comparative data indicate higher rates of anxiety and depressive disorders during 2020 compared with pre-pandemic periods, underscoring a macro-level rise in psychological distress that likely interacted with acute dental symptoms to depress QoL during pandemic peaks [17,18]. Population studies conducted during COVID-19 also documented shifts in affect and optimism across age groups, reflecting fluctuations in perceived well-being that may have amplified the impact of oral symptoms on QoL [19]. Cross-sectional studies from 2022 to 2025 consistently reported worse OHRQoL scores—most commonly measured using OHIP-14—in adults who experienced oral problems during COVID-19 waves, with psychological discomfort and social disability being among the most affected domains [20,21,22,23]. Convergent evidence from psycho-oncology further supports this pattern: a recent systematic review and meta-analysis showed that psychological distress is closely linked to poorer QoL in adult cancer patients, and that evidence-based non-pharmacological interventions can meaningfully reduce distress and improve QoL [24]. Recent studies from Iran and India similarly reported that post-COVID oral complaints, even relatively minor ones such as ulcers or halitosis, translated into measurable QoL deterioration, particularly in older or socio-economically vulnerable adults [20,21].

Despite this growing body of literature, several important gaps remain. First, most pandemic-era dental studies focused on service utilization rather than patient-reported outcomes, providing limited insight into how patients experienced and functioned during these events [25]. Second, QoL assessments in dentistry during COVID-19 relied predominantly on short OHIP instruments; comparatively less is known about the performance of broader, multidimensional QoL tools in acute dental conditions, despite methodological recommendations emphasizing the importance of sound psychometrics, clear scaling, and adequate domain coverage [17,18,26]. Third, very few studies have isolated the clinically most demanding scenario: patients who simultaneously present with a dental emergency and confirmed COVID-19. This combination is likely to exacerbate somatic symptoms, delay help-seeking, limit access to analgesic or antibiotic therapy, and increase anxiety related to attending dental facilities. Service reports suggest that these patients were among the most affected by postponements and limited availability of hospital-based dental care, yet QoL data for this group remain scarce [27,28].

Within the broader field of oral-health QoL research, several validated adult instruments are available, with established guidance for interpretation, longitudinal use, and cross-cultural adaptation. Systematic reviews indicate that instruments from the OHIP, OIDP, GOHAI, and related families can detect clinically meaningful changes following urgent dental treatment and correlate with pain, function, and mental-health indicators [5,17,29].

More recent work has called for context-sensitive tools or adapted batteries, particularly in acute conditions where symptoms may fluctuate rapidly. In this context, the 32-item Quality-of-Life Inventory (QOLI), which can be scored both as a continuous global index (SBQ) and as an ordinal global category (CGV, 0–3), offers a flexible framework that allows assessment of mean differences, ordered outcomes, and domain-level patterns within a single instrument. Psychometric research during and after the pandemic has further emphasized the importance of reporting effect sizes and confidence intervals, in addition to *p*-values, to improve the real-world magnitude of oral-health impacts [18,26].

In this study, the term “psycho-social impact” refers to the combined effects of acute dental pathology and pandemic-related stressors on psychological well-being and social functioning. Dental emergencies are frequently associated with intense pain, sleep disturbance, fear, anxiety, and emotional distress, which may be exacerbated by uncertainty related to SARS-CoV-2 infection, isolation measures, and concerns about accessing healthcare services. Socially, these conditions can interfere with daily activities, work performance, interpersonal relationships, and communication, while restrictions on routine dental care during the pandemic often limited timely access to professional support [30,31].

The present study was designed to compare COVID-19-positive adults presenting with a dental emergency with contemporaneous COVID-19-positive controls without urgent dental conditions, using the complete 32-item QOLI. The primary objective was to test the hypothesis that patients with dental emergencies would report significantly lower global QoL (SBQ) and would be shifted toward poorer QoL categories (CGV) than controls. A secondary objective was to explore whether specific QOLI domains—particularly those capturing pain interference, social or role limitation, and emotional distress—were disproportionately impaired in the emergency group after adjustment for age, sex, and available clinical covariates. By quantifying the magnitude of this QoL burden during COVID-19, the study provides patient-centered evidence relevant to clinical decision-making and to policy discussions on maintaining accessible emergency dental pathways during future public health crises [32,33,34].

## 2. Materials and Methods

### 2.1. Study Design and Setting

This investigation was designed as a cross-sectional case–control study, conducted at participating dental care sites in Romania between January 2022 and April 2024. Data collection took place at three locations in Galați city: the general medicine office (CMI Dr. Dragomir Gabriela), the dental office (Morodent Optimum SRL), and the Psychiatric Hospital “Elisabeta Doamna,” under standardized infection-prevention protocols applicable during the COVID-19 pandemic. Participants were recruited using a consecutive, non-probabilistic sampling strategy, enrolling all eligible individuals who presented to the participating sites during the study period.

The protocol was approved by the Ethics Committee of “Dunărea de Jos” University of Galați (No. 6/28 October 2024) and conducted in accordance with the Declaration of Helsinki. All participants provided written informed consent before any study procedures.

All participants provided written informed consent before inclusion. Demographic and contextual data (sex, age, recent dental visits, medication use, and systemic comorbidities, when available) were collected via standardized forms.

**QOLI Instrument and Scoring Algorithm**

The Quality-of-Life Inventory (QOLI) used in this study comprises 32 items, each rated on a 5-point Likert scale (1 = very dissatisfied to 5 = very satisfied). Items are grouped into four conceptual domains reflecting the respondent’s satisfaction and perceived importance across life areas relevant to oral and general health:

- Physical and functional well-being (e.g., pain, sleep, energy, daily activity).
- Psychological and emotional well-being (e.g., anxiety, self-image, mood stability);
- Social and relational functioning (e.g., family, friends, communication);
- Environment and access to care (e.g., dental services, financial constraints).

For each item, weighted satisfaction scores were computed as the product of

$$W_{k}=I_{k}\times S_{k}$$

where *I_k_* denotes importance (0 = not important, 1 = important, 2 = especially important) and *S_k_* denotes satisfaction recorded on the QOLI metric (−3 = very dissatisfied, −1 = dissatisfied, 0 = neutral, +1 = satisfied, +3 = very satisfied). Areas with *I_k_* = 0 were not included in the aggregation, consistent with QOLI conventions. Negative values indicate dissatisfaction in areas rated important to the participant.

The global QOLI score (SBQ) was calculated as the mean of all weighted item scores across valid responses (minimum 80% item completion required). Higher values denote a better perceived quality of life.

SBQ was defined as the arithmetic mean of all evaluable life areas (i.e., areas with *I_k_* > 0). Following psychometric guidance, a minimum of eight valid areas was required for SBQ to be scored; otherwise, SBQ was set to missing and excluded from inferential analyses.

Additionally, respondents were classified into an ordinal global quality-of-life category (CGV) as follows:

0—Very low quality of life: SBQ ≤ −2.0;

1—Low quality of life: −2.0 < SBQ ≤ 0;

2—Moderate quality of life: 0 < SBQ ≤ 2.0;

3—High quality of life: SBQ > 2.0.

Internal consistency reliability (Cronbach’s α) and domain-specific subscale scores were verified for both groups. Cases with >20% missing QOLI items were excluded from inferential analyses.

### 2.2. Participants

#### 2.2.1. Eligibility Criteria

Participants were adults aged 18 years or older who presented to the participating clinical sites in Galați, Romania, between January 2022 and April 2024. Inclusion required the ability to provide informed consent and to complete the 32-item QOLI questionnaire.

#### 2.2.2. Exclusion Criteria Were

- Cognitive impairment, acute psychiatric instability, or inability to understand the questionnaire items;
- Severe systemic illness (e.g., terminal cancer, advanced neurodegenerative disease) precluding valid self-reporting;
- Ongoing hospitalization or intensive care
- Missing responses in more than 20% of QOLI items.

#### 2.2.3. Group Definitions

Participants were categorized into two analytical groups:

**Case group** (Dental emergency + COVID-19): Patients presenting with a clinically confirmed dental emergency, defined as acute pain, infection, trauma, or hemorrhage requiring immediate intervention, and a concurrent or recent (≤14 days) positive SARS-CoV-2 test (RT-PCR or antigen) documented in their medical record or through national testing data.

**Control group** (Non-emergency during the COVID-19 period): Patients attending routine or scheduled dental visits without emergency dental conditions, recruited during the same COVID-19 pandemic period and from the same clinical settings as the case group.

Participants were not individually or frequently matched. Instead, a case–control design with contemporaneous recruitment was employed. Patients in both groups were enrolled during the same study period and under comparable pandemic-related conditions. Group comparability with respect to basic demographic characteristics was evaluated descriptively, and potential confounding by age and sex was addressed through multivariable statistical adjustment in all primary analyses.

Patients with confirmed SARS-CoV-2 infection (RT-PCR or antigen positivity within ≤14 days), recruited during the same pandemic period and from the same clinical settings as the case group, who did not present with a dental emergency or other acute dental pathology at the time of assessment.

### 2.3. Measures/Instruments

Sampling followed a consecutive, non-probabilistic approach, including all eligible individuals presenting during the specified period. Of the total screened participants, those meeting the inclusion criteria and consenting were enrolled and completed the QOLI instrument under research supervision in a dedicated consultation area.

The Quality-of-Life Inventory (QOLI) is a standardized instrument originally developed and psychometrically validated by Frisch et al. for the assessment of subjective quality of life across multiple life domains [35]. The instrument has demonstrated good internal consistency, construct validity, and sensitivity to change in both clinical and non-clinical populations.

For the present study, a Romanian-language version of the QOLI was used. This version was linguistically adapted following standard forward–backward translation procedures and was pretested for clarity and comprehension in a clinical sample before data collection. Internal consistency reliability (Cronbach’s α) was assessed within the current sample to confirm acceptable scale performance. No new questionnaire development or independent construct validation was undertaken by the authors; rather, the study relied on the established validation of the original instrument and its prior applications in health and dental research.

The final analytic sample comprised:

n_1_ = 60 in the case group.

n_2_ = 180 in the control group.

### 2.4. Variables

The main outcomes were derived from the Quality-of-Life Inventory (QOLI, thirty-two items), which provided both continuous and categorical indicators of well-being. The primary outcome was the global QOLI continuous score (SBQ), calculated as the mean of all valid weighted item scores. Higher SBQ values indicated a better overall quality of life. As secondary outcomes, the study examined the global categorical quality-of-life index (CGV), an ordinal variable ranging from 0 (very low quality of life) to 3 (high quality of life), and, where item structure permitted, four domain-specific subscales reflecting physical–functional, psychological–emotional, social–relational, and environmental aspects of quality of life. The global categorical quality-of-life variable (CGV) was derived from the continuous QOLI global score (SBQ) and operationalized as an ordinal outcome with four ordered categories reflecting overall quality of life:

**0 = very low quality of life** (SBQ < −2.0);

**1 = low quality of life** (−2.0 < SBQ ≤ 0);

**2 = moderate quality of life** (0 < SBQ ≤ 2.0);

**3 = high quality of life** (SBQ > 2.0).

Higher CGV values indicate better perceived quality of life.

Covariates were specified a priori: sex (0/1), age (years), recent dental visits (any preventive/control visit in the prior 12 months, yes/no), medication use (any regular pharmacotherapy in the last 30 days, yes/no), and comorbidities when available (cardiovascular disease, asthma, diabetes; yes/no for each). COVID-19 status was defined as RT-PCR or antigen positivity within ≤14 days for both the experimental and control groups, as documented in clinical records. The experimental group additionally met criteria for a clinically confirmed dental emergency, whereas controls had no acute dental pathology at the time of assessment. Self-reported indicators of anxiety, sleep disturbance, and fatigue were collected as binary variables to characterize the psychosocial context of participants during the pandemic period. These variables were intended for descriptive and exploratory analyses and were not included in the primary adjusted regression models. This decision was made a priori, as such symptoms may plausibly function as mediators in the pathway between dental emergencies and quality-of-life impairment rather than as baseline confounders.

Exploratory analyses also considered self-reported indicators of anxiety, asthenia, or sleep disturbance, coded as binary covariates. Before inclusion in regression models, all variables were examined for missing data, normality, and multicollinearity to ensure the robustness of subsequent analyses.

The exposure of interest was the presence of a dental emergency during the COVID-19 pandemic period, rather than differential exposure to pandemic-related contextual stressors, which were common to both groups.

In addition to the primary and secondary quality-of-life outcomes, several contextual and symptom-related variables were recorded at baseline for descriptive and exploratory purposes. These included:

- **VST (Visit status):** categorical variable reflecting the type of dental visit at presentation (coded as 2 = emergency visit, 3 = urgent but non-emergency visit, 4 = routine/scheduled visit). This variable was used to characterize clinical presentation patterns across groups.
- **MDU** (Medication use): binary variable indicating regular use of any systemic medication in the 30 days preceding assessment (1 = no, 2 = yes). Medication use was considered a potential proxy for underlying chronic conditions.
- **AST** (Asthenia/fatigue): binary self-reported indicator of clinically relevant fatigue at the time of assessment (1 = no, 2 = yes), included due to its known association with both COVID-19 infection and quality-of-life impairment.
- **TST** (Sleep disturbance): binary self-reported indicator of sleep problems (1 = no, 2 = yes), reflecting a common psychosomatic symptom during acute illness and pandemic-related stress.
- **ANX** (Anxiety symptoms): binary self-reported indicator of anxiety or heightened nervousness at the time of assessment (1 = no, 2 = yes), included given its relevance to psychosocial functioning and quality-of-life outcomes.

These variables were primarily used for descriptive comparisons between groups and exploration analyses, rather than as primary predictors, and were coded uniformly across study sites using standardized data collection forms.

COVID-19 status was defined as RT-PCR or antigen positivity within ≤14 days and constituted an inclusion criterion for both study groups. The exposure of interest was the presence of a dental emergency among COVID-19-positive patients.

### 2.5. Data Sources/Measurement

The Quality-of-Life Inventory (QOLI) used in this study consisted of thirty-two self-report items assessing satisfaction and importance across key life domains relevant to oral and general health. The Romanian version of the instrument was linguistically adapted and pretested for clarity and internal consistency within a clinical population, following the principles of forward–backward translation and conceptual validation recommended by the World Health Organization. Its structure and psychometric properties are consistent with validated QOLI applications in health and dental research. For exploratory comparisons and visualizations, domain-level scores were also calculated by averaging the corresponding weighted items within each theoretical subscale: physical–functional, psychological–emotional, social–relational, and environmental well-being.

Item-level reverse scoring was applied to negatively phrased items before aggregation to ensure consistent directionality (e.g., items related to pain, fatigue, or anxiety). Where applicable, continuous SBQ values were rescaled to a 0–one hundred range for comparability with other quality-of-life instruments, using a linear transformation based on the observed minimum and maximum.

Missing data were examined at the item level. Participants with more than 20% missing QOLI responses were excluded from the analytic sample. For cases with minor missingness (≤10%), scores were computed using available items without imputation, as missingness was assumed to be random and limited in scope. No statistical imputation was performed unless explicitly justified in sensitivity analyses.

### 2.6. Bias and Study Size

To minimize potential sources of bias, several methodological precautions were implemented throughout the study. Selection bias was reduced by employing a consecutive recruitment strategy, enrolling all eligible patients who presented to the clinic during the predefined data collection period. Both groups were drawn from the same clinical setting and pandemic context, ensuring comparable environmental, social, and epidemiological conditions. The same research staff applied the inclusion and exclusion criteria uniformly to avoid subjective screening discrepancies.

Information bias was addressed by using a standardized administration protocol for the QOLI instrument. Participants completed the questionnaire under research supervision in a quiet consultation area, and clarifications were provided only in neutral, non-leading terms to maintain consistency. Data entry and coding were double-checked by two independent investigators, and automated algorithms were applied for scoring and data cleaning to minimize transcription errors.

Given the observational nature of the study, blinding was not applicable for exposure status (dental emergency vs. control). However, QOLI data were self-reported and analyzed anonymously, independent of clinical identifiers, which limited differential reporting bias.

The sample size was determined by the availability of eligible participants within the study period rather than by a priori power calculation, consistent with the pragmatic design of pandemic-era research. The final dataset comprised 240 participants (60 in the case group and 180 in the control group), which provided adequate power (>0.80) to detect a medium standardized effect size (Cohen’s d ≈ 0.5) for between-group differences in the primary outcome (SBQ), assuming a two-tailed α of 0.05 and unequal group sizes (n_1_:n_2_ = 1:3).

Regarding sex distribution, the control group comprised 80 females (44.4%) and 100 males (55.6%), while the case group included 19 females (31.7%) and 41 males (68.3%). No statistically significant difference in sex distribution was observed between groups (χ^2^(1) = 2.53, *p* = 0.112), indicating comparable representation by sex across study groups.

Post-hoc sensitivity analyses confirmed that the sample was sufficient to detect moderate effects on both continuous and ordinal outcomes, while smaller effects (d < 0.3) would require larger samples for conclusive detection.

### 2.7. Statistical Methods

Descriptive analyses were first conducted to summarize demographic, clinical, and outcome variables. Continuous variables were expressed as mean ± standard deviation (SD) for normally distributed data or as median [interquartile range, IQR] when deviations from normality were observed, as assessed by the Shapiro–Wilk test and visual inspection of histograms and Q-Q plots. Categorical variables were reported as absolute frequencies (n) and percentages (%).

For between-group comparisons, the global QOLI continuous score (SBQ) served as the primary dependent variable. Differences between the experimental and control groups were evaluated using Welch’s two-sample *t*-test for unequal variances; when normality assumptions were not met, the Mann–Whitney U test was applied. Effect sizes were reported as Cohen’s d (or Hedges’ g to account for unequal sample sizes), together with 95% confidence intervals (CI) and two-tailed *p* values.

The ordinal global quality-of-life category (CGV) was derived from SBQ using fixed cut-points: 0 = very low (SBQ < −2.0), 1 = low (−2.0 < SBQ ≤ 0), 2 = moderate (0 < SBQ ≤ 2.0), and 3 = high (SBQ > 2.0). This rule is consistent with prior QOLI applications and aligns with the observed sample distributions.

For categorical covariates (e.g., medication use, recent dental visits, or presence of comorbidities), differences between groups were evaluated with Pearson’s χ^2^ test or Fisher’s exact test, as appropriate. Where relevant, risk ratios (RR) or odds ratios (OR) were presented alongside 95% CIs to quantify effect magnitudes.

The primary adjusted analyses employed multivariable models to control for potential confounders.

For the continuous outcome (SBQ), we fitted a linear model with robust HC3 standard errors:

$$SBQ=\beta_{0}+\beta_{1}(group)+\beta_{2}(sex)+\beta_{3}(age)+\beta_{k}(covariates)+\varepsilon$$

With heteroskedasticity-consistent robust standard errors (HC3) to ensure reliable inference.

For the ordinal outcome (*CGV*), a proportional-odds logistic regression was specified:

$$logit[P(CGV\geq j)]=\alpha_{j}-\beta_{1}(group)-\beta_{2}(sex)-\beta_{3}(age)-\beta_{k}(covariates),$$

Retaining the proportional-odds structure unless assumption checks indicated otherwise.

For exploratory comparisons of QOLI subdomains, *p*-values were corrected for multiple testing using the False Discovery Rate (FDR) procedure.

**Sensitivity analyses** were conducted by repeating all primary tests after 5% winsorization of SBQ scores to attenuate the influence of outliers. The robustness of conclusions was evaluated by examining the consistency of effect directions, magnitudes, and significance levels. After 5% winsorization of SBQ, the direction and magnitude of the group effect were unchanged across all tests and models (unadjusted and adjusted), indicating that results were not driven by extreme values.

Multicollinearity among predictors in the adjusted regression models was evaluated using variance inflation factors (VIF). All VIF values for included covariates were below 5, indicating no evidence of problematic multicollinearity.

All analyses were performed in R version 4.5.0 (R Foundation for Statistical Computing, Vienna, Austria) using the tidyverse, rstatix, effectsize, sandwich, and brant packages for proportional-odds diagnostics. In the absence of formal matching, potential confounding by demographic variables was addressed using multivariable regression models. Given the unequal group sizes, results are primarily interpreted based on standardized effect sizes and corresponding 95% confidence intervals, rather than sole reliance on null-hypothesis significance testing. To minimize the risk of overadjustment, only demographic variables (age and sex) were included as covariates in the primary models, whereas psychosocial symptom indicators were examined descriptively and in sensitivity analyses.

Sensitivity analyses excluding psychosocial symptom indicators (anxiety, sleep disturbance, fatigue) from all models yielded effect estimates that were materially unchanged, indicating that the primary findings were robust to alternative covariate specifications.

## 3. Results

### 3.1. Participant Flow and Sample

A consecutive series of eligible patients was approached during the study period; pre-consent screening counts were not recorded in the exported registries. No post-consent exclusions occurred; 0/240 cases exceeded the missing-data threshold, and both SBQ and CGV were complete.

Final sample: The final analytic sample comprised *n* = 240 participants: control group *n* = 180 (75.0%) and case group *n* = 60 (25.0%).

Data completeness (quality check).

QOLI items (32 per participant): Control—1 missing item in total (1/180 participants had ≥1 missing item; 0.02% item-level missingness overall); Experimental—0 missing items. No participant exceeded the predefined threshold of >20% missing QOLI items; thus, 0/240 were excluded for missingness. SBQ and CGV were complete (0/240 missing).

SBQ and CGV: 0/240 missing values; all participants contributed to primary and secondary outcomes.

### 3.2. Descriptive Data

A total of 240 participants were analyzed (Control: 180; Experimental: 60). Baseline characteristics and between-group comparisons are summarized in Table 1, with standardized effect sizes and confidence intervals reported to facilitate interpretation. In brief, sex distribution and other recorded contextual variables showed no large imbalances. Unadjusted QOLI outcomes showed substantial between-group differences, consistent with the study hypothesis.

For the global QOLI score (SBQ), the Control group had a mean ± SD of 0.82 ± 1.44 (*n* = 180), whereas the case group had −1.22 ± 1.10 (*n* = 60). The unadjusted mean difference was Δ = −2.04 (95% CI −2.39 to −1.69), indicating notably worse quality of life in patients with dental emergencies and COVID-19. The standardized effect size was large (Hedges’ g = −1.49, 95% CI −1.81 to −1.17). Between-group tests and effect sizes for all baseline variables are reported in Table 1.

### 3.3. Primary and Secondary Outcomes

**Figure 1:**
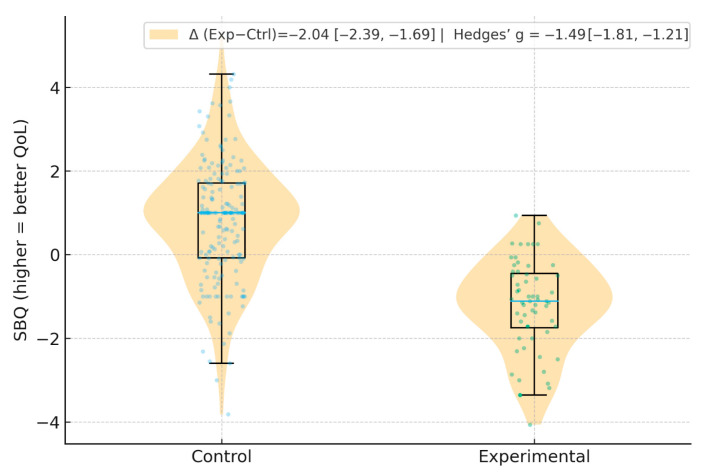
SBQ distributions by group. Higher SBQ scores indicate a better quality of life. The case group shows a clear leftward shift. Annotated: unadjusted mean difference (Δ, Experimental—Control) and Hedges’ g with 95% bootstrap CIs (10,000 resamples).

**Primary outcome (SBQ, continuous):** SBQ was lower in the case group (Δ_{Exp–Ctrl} = −2.04, 95% CI −2.39 to −1.69; Hedges’ g = −1.49, 95% CI −1.81 to −1.21; Figure 1).

**Secondary outcome (CGV, ordinal 0–3):** The unadjusted distribution favored higher (better) categories in controls and lower (worse) categories in the case group (see Table 1). Model-based estimates from the planned ordinal logistic analyses (both unadjusted and adjusted) are presented in the Outcomes Analysis section, along with the corresponding results tables.

Findings were consistent in adjusted analyses: the group effect remained large and negative for SBQ (Model B), and the odds of being in a better CGV category were markedly lower in the case group (Model D). Proportional-odds diagnostics did not indicate meaningful violations; thus, the PO specification was retained.

### 3.4. Exploratory Analyses

#### Domain/Subscale Contrasts (QOLI)

Each QOLI domain was scored as importance × satisfaction, where paired items (Q1–Q32) were mapped to 16 life areas. Between-group contrasts used Welch’s t-tests with Hedges’ g (95% CI). Multiplicity was controlled with Benjamini–Hochberg FDR across the sixteen domains.

**Overall pattern:** In the case group, 13/16 domains were significantly worse after FDR (q < 0.05). The largest standardized gaps were in Play (g ≈ −0.87), Work (g ≈ −0.74), Self-esteem (g ≈ −0.72), Creativity (g ≈ −0.67), and Goals and values (g ≈ −0.65). Three domains were not significant after FDR: Relatives (q = 0.877), Neighborhood (q = 0.598), and Community (q = 0.135).

**Interpretation:** Beyond the global between-group difference in QOLI reported in Table 1, participants with dental emergency + COVID-19 showed broad, multi-domain decrements in quality of life. The largest domain-level impairments were observed in play/leisure, work functioning, and self-evaluation/values, indicating that the impact of acute dental pathology during COVID-19 extends from symptoms to role performance and core self-appraisal (Table 2). 

### 3.5. Sensitivity Analyses (Outlier Handling)

We repeated domain comparisons after 5% winsorization of each domain score (pooled), then re-ran Welch tests, Hedges’ g, and BH-FDR.

Findings. Conclusions were unchanged: 13/16 domains remained significant after FDR; no domain changed significance status. Effect sizes were stable (max |Δg| ≈ 0.13; mean |Δg| ≈ 0.02), with the largest shift for Play (g from −0.87 to −1.00).

### 3.6. Additional Analysis

**Rationale and scope:** Subgroup and robustness checks were treated as exploratory, given the study’s prespecified primary objective and the unequal sample sizes (*n* = 60 vs. *n* = 180). We aimed to determine whether the main group effect (Emergency COVID vs. Control) on QOLI outcomes varied across simple strata and whether conclusions were sensitive to analytic choices.

**Subgroup checks**

We assessed whether the between-group difference varied by sex by adding an interaction term (Group × Sex) to the primary models (SBQ: linear regression with HC3 robust SE; CGV: proportional-odds ordinal logistic). Interaction terms did not provide a credibly different pattern from the overall effect; stratum-specific contrasts were directionally consistent with the main analysis and of similar magnitude. In keeping with STROBE, we therefore present inference on the overall effect and label these checks as exploratory.

Model diagnostics and alternative estimators.

**Heteroskedasticity and influence:** SBQ models were fit with HC3 robust SE; influence diagnostics (fitted vs. residuals, leverage/DFBETAs) did not reveal single-case dependence sufficient to overturn conclusions.

**Non-normality:** As a distribution-agnostic alternative, we repeated the SBQ contrast using a Mann–Whitney test and a median (quantile) regression model; both yielded the same qualitative conclusion (worse SBQ in the case group).

**Figure 2:**
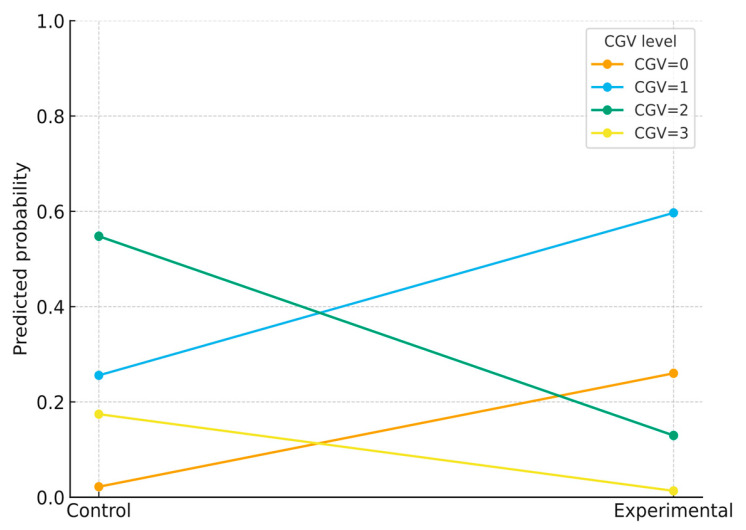
Model-predicted probabilities for CGV (0–3) by group from a proportional-odds ordinal logistic model (covariates: sex). Points show the predicted probability at the mean of covariates; lines connect group levels. Higher CGV indicates a better quality of life.

**Ordinal outcome assumption:** For CGV, the proportional-odds assumption was checked; no material violations were detected. A partial proportional odds fit produced estimates aligned with the main model. The case group had markedly lower odds of being in a better CGV category compared with controls (OR = 0.064, 95% CI 0.032–0.129; proportional-odds model adjusted for sex; Figure 2). Model-predicted probabilities (at mean sex) were controls CGV = 0: 0.022, CGV = 1: 0.256, CGV = 2: 0.548, CGV = 3: 0.174; experimental CGV = 0: 0.260, CGV = 1: 0.597, CGV = 2: 0.130, CGV = 3: 0.013.

#### Sensitivity to Extreme Values

Because acute presentations can generate outliers, we repeated the analyses after 5% winsorization of QOLI scores. Effect directions and interpretation were unchanged; the magnitude of standardized effects varied only trivially (the largest absolute shift was small; the mean shift was negligible).

Across exploratory strata and robustness choices, the main conclusion holds that patients with dental emergencies during COVID-19 report poorer quality of life. Given their post-hoc and underpowered nature, these checks are reported for completeness rather than for confirmatory inference.

### 3.7. Clinical Interpretation of the Main Findings

From a clinical perspective, the results indicate that COVID-19-positive patients presenting with a dental emergency experienced a markedly poorer overall quality of life compared with contemporaneous COVID-19-positive patients without urgent dental conditions. In practical terms, patients in the emergency group reported substantially greater impairment in daily functioning, including more intense pain, greater sleep disturbance, heightened emotional distress, and increased difficulty performing usual activities. These impairments were not marginal but reflected a profound deterioration in perceived well-being, consistent with clinical presentations characterized by severe discomfort and functional limitation.

Importantly, the shift toward lower quality-of-life categories observed among emergency patients suggests that dental emergencies during COVID-19 were associated not only with transient symptoms but also with a broader disruption of psychological balance and social functioning. Clinically, this translates into patients being more likely to experience difficulties in work performance, social interaction, and communication, along with increased anxiety and reduced coping capacity. In contrast, COVID-19-positive patients without dental emergencies generally reported preserved or only moderately affected quality of life, highlighting the specific and additive burden imposed by acute dental pathology in the pandemic context.

Overall, these findings underscore that dental emergencies during COVID-19 represent more than isolated oral health events; they constitute complex clinical situations with significant psycho-social consequences that may require integrated pain management, psychological support, and timely access to emergency dental care to mitigate their impact on patients’ quality of life.

## 4. Discussion

Given the cross-sectional design, the observed differences in quality of life should be interpreted as associations rather than evidence of causal relationships between dental emergencies, SARS-CoV-2 infection, and psychosocial outcomes.

### 4.1. Principal Findings

This cross-sectional case–control study found that emergency dental patients with recent or current COVID-19 experienced a significant decrease in overall quality of life (SBQ) and had notably lower chances of being in higher CGV categories compared to controls. Domain-specific analysis revealed broad declines, especially in areas such as play/leisure, work, self-esteem, creativity, and goals/values. All participants in both groups confirmed they had SARS-CoV-2 infection. Consequently, the study focuses on exploring the relationship between dental emergency status and quality of life among COVID-19-positive individuals, rather than comparing COVID-19-positive and COVID-19-negative groups.

### 4.2. Interpretation and Clinical Relevance

Dental emergencies combine acute nociceptive/inflammatory pain, functional impairment (chewing, sleep), and psychological distress, known to reduce quality of life and daily functioning. Timely intervention for acute dental pain improves patient-reported outcomes and prevents complications, underscoring the clinical importance of preserving urgent pathways even under system strain [36]. COVID-19 service restrictions (emergency-only care, stringent IPC/PPE measures) plausibly intensified access barriers and delayed presentations, amplifying pain and psychosocial burden [37,38,39]. Converging evidence documents declines in oral health perceptions and OHRQoL during the pandemic and slow recovery of dental service use relative to other care, supporting the context in which our cohort was treated [1,40]. Beyond orofacial symptoms, COVID-19 has had persistent mental-health sequelae for a subset of patients, which can further depress quality of life and coping mechanisms consistent with our global QOLI deficits [41]. Aligned with this, psycho-oncology meta-analytic evidence indicates that structured, non-pharmacological interventions improve patient-reported QoL by alleviating distress; by analogy, embedding brief psychological support alongside urgent dental care may offer incremental benefits during crises [24].

At a mechanistic level, acute dental pain disrupts sleep and work, limits nutrition, and restricts social participation—well-characterized pathways through which oral conditions degrade overall functioning [42,43]. Our findings that the largest QOLI gaps were in play/leisure, work, self-esteem, creativity, and goals/values are consistent with this broader biopsychosocial impact, not merely mouth-specific symptoms [42].

An additional contextual factor relevant to these findings is the occupational exposure of dental healthcare professionals to SARS-CoV-2. Owing to close face-to-face contact and frequent aerosol-generating procedures, dental professionals were considered a high-risk occupational group during the pandemic, necessitating stringent infection-prevention measures and, at times, restriction of routine care. Evidence indicates that such exposure was associated not only with increased infection risk but also with heightened psychological burden among dental staff, which may have indirectly influenced service availability and patients’ perceptions of safety in dental settings during COVID-19 [44,45,46].

Vaccination against SARS-CoV-2 emerged as a key protective strategy for dental professionals and patients, contributing to reduced disease severity and supporting continuity of essential dental services. However, concerns regarding vaccine safety, side effects, and evolving recommendations persisted in both the general population and healthcare workforce, potentially shaping trust in healthcare environments and willingness to seek in-person care. Balancing the clear public-health benefits of vaccination with transparent risk communication appears critical for maintaining patient confidence and access to urgent dental care during future infectious disease outbreaks.

### 4.3. Comparison with Prior Literature

Pre-/post-intervention studies in urgent dental care centers show meaningful reductions in OHIP scores after emergency treatment, indicating rapid, patient-perceived benefits; our cross-sectional contrasts point in the same direction from a general QoL perspective (QOLI rather than OHRQoL) [5]. Community and hospital-based work consistently associates dental pain with impaired daily activities and poorer well-being, mirroring our domain-level decrements [43,47]. During pandemic phases, routine dental activity contracted markedly, ED attendances for orofacial pain/infection rose, and oral/maxillofacial surgery services were curtailed, patterns that plausibly increased unmet need and symptom severity in “emergency” case mix [48,49]. Broader reviews link oral health to general health and QoL across the life course, strengthening the rationale for safeguarding urgent dental access even amidst system shocks [42,50,51].

Case-based evidence also illustrates bidirectional links between depressive disorders and deteriorating oral status; a persistent depressive disorder case documented resistant depression alongside marked dental pathology and functional impairment, highlighting plausible behavioral pathways (reduced self-care, delayed care-seeking) relevant to our cohort [52].

Although OHRQoL instruments (e.g., OHIP) dominate dental outcomes research, the QOLI is a validated 32-item, life-domain inventory capturing broader life satisfaction and importance weighting; using it here complements OHRQoL by quantifying whole-person impact [53,54].

### 4.4. Strengths and Limitations

As this study is cross-sectional, temporal ordering between dental emergencies, COVID-19 infection, and quality-of-life outcomes cannot be established, and causal interpretations should be avoided.

Strengths include a clear a priori contrast (emergency COVID vs. contemporaneous controls), use of a validated QoL instrument (QOLI), transparent reporting per STROBE, and effect-size-centric inference with uncertainty (CIs) plus multiplicity control (BH-FDR) [55]. Robustness checks (HC3 SE; winsorization) and assumption checks for ordinal modeling increase credibility [56].

Several limitations should be considered when interpreting these findings. First, the cross-sectional design precludes any inference regarding causal direction or temporal ordering between dental emergencies and quality-of-life outcomes. Second, the study relied on a consecutive, non-probabilistic sample drawn from a limited number of clinical sites. Although this pragmatic approach was appropriate under pandemic-related constraints, it introduces potential selection bias and limits external validity. Pandemic-era service reconfiguration and infection prevention and control (IPC) protocols may therefore restrict the generalizability of the results to settings operating under similar conditions [37].

Pre-consent screening and eligibility counts, including refusal rates, were not systematically recorded, precluding the reconstruction of a complete recruitment flow. This limitation prevents a formal assessment of participation or non-response bias. Nevertheless, missingness in primary and secondary outcomes was zero, item-level missingness was negligible, and outcome data were handled according to a predefined analytic plan. Despite these safeguards, residual confounding due to unmeasured factors such as socioeconomic stressors, comorbidity burden, or individual care-seeking thresholds cannot be fully excluded.

Another limitation relates to outcome measurement. Oral health-specific quality of life (OHRQoL) was not directly assessed using dedicated dental instruments. However, substantial prior evidence demonstrates strong associations between oral symptoms (pain, infection, functional limitation) and daily functioning, psychological distress, and social participation, which are well captured by global quality-of-life measures and triangulate with the observed QOLI findings [47].

A further limitation concerns the temporal relationship to SARS-CoV-2 infection. While recency of infection (≤14 days) was explicitly defined and documented for the experimental group, prior COVID-19 history among control participants was not systematically captured. Because recent or prior infection may independently influence psychological distress and perceived quality of life, residual heterogeneity related to past infection status among controls cannot be excluded. Nonetheless, all participants were assessed during the same pandemic waves and under comparable contextual stressors, and the large and consistent effect sizes observed across unadjusted, adjusted, and sensitivity analyses suggest that the primary findings are robust to this limitation.

Psychosocial symptoms such as anxiety, sleep disturbance, and fatigue were assessed using binary self-report items rather than validated scales with severity grading. This approach may introduce measurement error and misclassification and limit a more nuanced interpretation of symptom burden. These variables were therefore not treated as primary confounders in adjusted analyses. Future studies should incorporate validated psychosocial instruments and explicit causal frameworks to better distinguish confounding from mediation.

Finally, the absence of direct socioeconomic measures—such as income, education, employment status, or structural access barriers—represents an important limitation. In quality-of-life research, these factors are central determinants of both healthcare utilization and perceived well-being. Their omission introduces the possibility of residual confounding, as socioeconomic stressors may influence both the likelihood of presenting with a dental emergency during the pandemic and the magnitude of reported quality-of-life impairment. Consequently, the present findings cannot determine whether the observed decline in quality of life was uniformly distributed across the population or disproportionately concentrated among socioeconomically vulnerable subgroups.

As a single-city study, the results should therefore be interpreted as internally valid associations within a specific healthcare and pandemic context rather than as population-level estimates. Future multi-center and longitudinal studies incorporating detailed socioeconomic indicators, validated OHRQoL instruments, and formal recruitment flow documentation are warranted to further strengthen causal inference and generalizability.

### 4.5. Implications for Practice and Policy

Even during widespread health system disruptions, maintaining accessible and protocolized urgent dental care pathways is likely to yield substantial patient-important gains in quality of life, reduce inappropriate emergency department utilization, and prevent downstream complications [5]. The present findings support policy approaches that classify urgent dental care as an essential health service during pandemics, rather than a deferrable elective activity.

At the system level, pandemic preparedness plans should explicitly incorporate emergency dental services within continuity-of-care frameworks, including infection-prevention protocols, referral networks, and coordination with primary care and mental health services. Guidance from professional bodies such as the ADA and ECDC on emergency-first service models and infection prevention and control can inform standardized workflows during future pandemic waves or comparable crises [37,38,39].

Given well-documented social gradients in the burden and costs of oral pain, equity-focused strategies, such as targeted outreach, expedited referral pathways, and prioritization of high-risk populations, should be integral to policy planning to mitigate disproportionate psycho-social and quality-of-life impacts [57].

### 4.6. Clinical and Practical Implications

From a clinical perspective, the marked deterioration in quality of life observed among patients presenting with dental emergencies underscores the importance of timely access to definitive emergency dental treatment to alleviate not only pain and infection but also associated psycho-social distress. Delays in care during the COVID-19 pandemic have been associated with more invasive procedures and increased psychological stress, reinforcing the need for rapid and structured access pathways in crisis settings [58,59].

Integrating patient-reported outcome measures (PROMs), such as quality-of-life tools, into emergency dental triage may help clinicians identify patients at heightened risk of severe psycho-social impact and functional impairment. Such tools can support more patient-centered decision-making and facilitate the prioritization of supportive interventions alongside clinical treatment. Evidence from emergency dental care contexts indicates that PROMs are capable of capturing meaningful changes in oral health-related quality of life and patient satisfaction, supporting their utility for real-time service evaluation and quality improvement during health system disruptions [5].

In addition, brief, structured, non-pharmacological psycho-social interventions may represent a feasible adjunct to emergency dental care during pandemics, particularly for patients experiencing high levels of anxiety or distress. Embedding such supportive measures within urgent care workflows could contribute to reducing the cumulative psycho-social burden associated with dental emergencies under crisis conditions [24,33].

Beyond individual clinical factors, the psychosocial burden associated with dental emergencies during the COVID-19 period should be considered within broader social determinants of health. Pandemic-related stressors such as socioeconomic disadvantages, restricted access to care, and contextual vulnerability have been associated with increased psychological distress and poorer quality-of-life outcomes in diverse populations during COVID-19. Evidence indicates that economic hardship and labor market disruptions were linked to heightened psychosocial risk during the pandemic, reflecting how structural factors intersect with mental health and well-being [60,61,62]. These social and economic gradients may amplify the association between acute oral health needs and perceived quality-of-life impairment, particularly among individuals facing greater socioeconomic stress. Although socioeconomic variables were not directly measured in the present study, recognizing this broader context underscores the public-health relevance of accessible emergency dental care and psychosocial support during periods of systemic disruption.

### 4.7. Future Research

Prospective cohort or stepped-wedge designs comparing alternative emergency pathways could estimate causal effects on global QoL and OHRQoL, service use, and costs. Studies should integrate repeated measures pre-/post-intervention (including pain, sleep, anxiety), explore mechanistic mediators, and incorporate cost-effectiveness. When modeling ordinal outcomes (e.g., global categories), proportional-odds frameworks-with diagnostics and partial PO relaxations as needed-remain appropriate; multiplicity should be controlled (e.g., BH-FDR) in multi-domain analyses [57,63].

## 5. Conclusions

In this cross-sectional, case–control study, patients with dental emergencies and COVID-19 reported lower quality of life than controls, with a global SBQ deficit of −2.04 points (95% CI −2.39 to −1.69) and a large, standardized effect. On the ordinal outcome, the odds of being in a better QoL category were ~93–94% lower in the emergency group (OR ≈ 0.065), confirming a marked decrement in self-reported well-being. These effects were robust to alternative analyses and model-assumption checks. The QoL decline was broad and multidomain (significant in 13/16 QOLI areas), underscoring the biopsychosocial impact of dental emergencies in a pandemic context. These findings support maintaining rapid, safe pathways to emergency dental care during crises-standardized triage, prompt pain/infection control, and integrated psychological support-while prospective studies are needed to assess the causal effects of service models on patient-reported quality of life.

Dental emergencies occurring during the COVID-19 pandemic were strongly associated with poorer patient-reported quality of life, highlighting the need for accessible emergency dental care and supportive interventions during periods of health system disruption.

## Figures and Tables

**Table 1:** Baseline characteristics and unadjusted outcome distributions by group.

Variable	Control (*n* = 180)	Experimental (*n* = 60)	Test	*p*-Value	Effect Size	95% CI
SBQ, mean ± SD	0.82 ± 1.44	−1.22 ± 1.10	Welch *t*	<0.001	Hedges’ *g* = −1.49	−1.81 to −1.17
Sex (female), *n* (%)	80 (44.4)	19 (31.7)	χ^2^	0.112	φ = 0.10	—
Sex (male), *n* (%)	100 (55.6)	41 (68.3)	χ^2^	0.112	φ = 0.10	—
Vaccination status—unvaccinated (VST = 2), *n* (%)	5 (2.8)	3 (5.0)	χ^2^	0.567	φ = 0.07	—
Vaccination status—partially vaccinated (VST = 3), *n* (%)	21 (11.7)	5 (8.3)	χ^2^	0.567	φ = 0.07	—
Vaccination status—fully vaccinated (VST = 4), *n* (%)	154 (85.6)	52 (86.7)	χ^2^	0.567	φ = 0.07	—
Recent medication use—no (MDU = 1), *n* (%)	134 (74.4)	42 (70.0)	χ^2^	0.613	φ = 0.03	—
Recent medication use—yes (MDU = 2), *n* (%)	46 (25.6)	18 (30.0)	χ^2^	0.613	φ = 0.03	—
Asthenia/fatigue—no (AST = 1), *n* (%)	12 (6.7)	6 (10.0)	χ^2^	0.571	φ = 0.04	—
Asthenia/fatigue—yes (AST = 2), *n* (%)	168 (93.3)	54 (90.0)	χ^2^	0.571	φ = 0.04	—
Sleep disturbance—no (TST = 1), *n* (%)	7 (3.9)	1 (1.7)	χ^2^	0.678	φ = 0.03	—
Sleep disturbance—yes (TST = 2), *n* (%)	173 (96.1)	59 (98.3)	χ^2^	0.678	φ = 0.03	—
Anxiety symptoms—no (ANX = 1), *n* (%)	86 (47.8)	21 (35.0)	χ^2^	0.115	φ = 0.10	—
Anxiety symptoms—yes (ANX = 2), *n* (%)	94 (52.2)	39 (65.0)	χ^2^	0.115	φ = 0.10	—
CGV = 0, *n* (%)	6 (3.3)	14 (23.3)	χ^2^	<0.001	φ = 0.55	—
CGV = 1, *n* (%)	44 (24.4)	39 (65.0)	χ^2^	<0.001	φ = 0.55	—
CGV = 2, *n* (%)	98 (54.4)	7 (11.7)	χ^2^	<0.001	φ = 0.55	—
CGV = 3, *n* (%)	32 (17.8)	0 (0.0)	χ^2^	<0.001	φ = 0.55	—

Notes: Between-group comparisons were performed using Welch’s *t*-test for continuous variables and χ^2^ tests for categorical variables. Effect sizes are reported as Hedges’ *g* for continuous outcomes and φ coefficients for categorical outcomes, with 95% confidence intervals where applicable. SEX: 0 = female, 1 = male; VST = vaccination status against COVID-19 (2 = unvaccinated, 3 = partially vaccinated, 4 = fully vaccinated); MDU = regular medication use in the last 30 days (1 = no, 2 = yes); AST = self-reported asthenia/fatigue (1 = no, 2 = yes); TST = sleep disturbance (1 = no, 2 = yes); ANX = self-reported anxiety symptoms (1 = no, 2 = yes). Participants were not individually or frequency matched. SBQ and CGV are presented descriptively to illustrate unadjusted between-group differences and are not considered baseline matching variables. Group coding: 0 = control, 1 = experimental. Confidence intervals are reported for continuous outcomes; for categorical effect size measures (φ), confidence intervals are not routinely reported and are therefore omitted.

**Table 2:** Domain-specific quality-of-life differences between control participants and patients with dental emergencies and COVID-19.

Domain	Control (Mean ± SD)	Experimental (Mean ± SD)	Mean Difference [95% CI]	Hedges g [95% CI]	*p*-Value	FDR q (BH)
Health	3.57 ± 2.27	2.28 ± 2.08	−1.29 [−1.92, −0.66]	−0.58 [−0.87, −0.28]	<0.001	<0.001
Self-esteem	3.17 ± 2.19	1.68 ± 1.61	−1.48 [−2.01, −0.96]	−0.72 [−1.02, −0.42]	<0.001	<0.001
Goals and values	2.97 ± 2.25	1.58 ± 1.63	−1.39 [−1.92, −0.86]	−0.65 [−0.95, −0.36]	<0.001	<0.001
Money	2.90 ± 1.83	1.92 ± 1.86	−0.98 [−1.53, −0.43]	−0.53 [−0.83, −0.24]	<0.001	<0.001
Work	2.65 ± 1.79	1.33 ± 1.75	−1.32 [−1.84, −0.80]	−0.74 [−1.04, −0.44]	<0.001	<0.001
Play and leisure	2.46 ± 1.93	0.90 ± 1.28	−1.56 [−2.00, −1.13]	−0.87 [−1.17, −0.56]	<0.001	<0.001
Learning	2.93 ± 2.23	1.77 ± 1.86	−1.16 [−1.74, −0.58]	−0.54 [−0.84, −0.24]	<0.001	<0.001
Creativity	2.86 ± 2.52	1.27 ± 1.93	−1.59 [−2.21, −0.98]	−0.67 [−0.96, −0.37]	<0.001	<0.001
Helping others	2.59 ± 1.95	1.60 ± 1.62	−0.99 [−1.50, −0.49]	−0.53 [−0.83, −0.23]	<0.001	<0.001
Love	3.33 ± 2.16	2.03 ± 2.44	−1.30 [−2.00, −0.60]	−0.58 [−0.88, −0.28]	<0.001	<0.001
Friends	2.57 ± 1.99	1.67 ± 1.65	−0.90 [−1.42, −0.38]	−0.47 [−0.76, −0.17]	0.001	0.001
Children	4.19 ± 3.32	3.23 ± 2.88	−0.96 [−1.85, −0.08]	−0.30 [−0.59, −0.00]	0.033	0.041
Relatives	1.23 ± 1.50	1.27 ± 1.42	0.03 [−0.39, 0.46]	0.02 [−0.27, 0.31]	0.877	0.877
Home	4.32 ± 2.88	2.72 ± 2.52	−1.60 [−2.37, −0.83]	−0.57 [−0.87, −0.27]	<0.001	<0.001
Neighborhood	1.16 ± 1.56	1.03 ± 1.35	−0.12 [−0.54, 0.29]	−0.08 [−0.37, 0.21]	0.561	0.599
Community	1.16 ± 1.55	0.83 ± 1.34	−0.33 [−0.74, 0.08]	−0.22 [−0.51, 0.08]	0.118	0.135

Note: Welch’s *t*-tests; Hedges’ g with 95% CI; Benjamini–Hochberg FDR across 16 domains.

## Data Availability

Due to the sensitive nature of clinical data, the dataset generated and analyzed during the current study is not publicly available.

## Abbreviations

The following abbreviations are used in this manuscript:

COVID-19	Coronavirus Disease 2019
CGV	Categorical Global Value (QOLI global ordinal quality-of-life category)
QOLI	Quality-of-Life Inventory (32-item instrument)
SBQ	Summary/Global QOLI Score (continuous)
QoL	Quality of Life
OHRQoL	Oral Health-Related Quality of Life
OHIP	Oral Health Impact Profile
RT-PCR	Reverse Transcription Polymerase Chain Reaction
OR	Odds Ratio
CI	Confidence Interval
SD	Standard Deviation
IQR	Interquartile Range
FDR	False Discovery Rate
HC3	Heteroskedasticity-Consistent Standard Errors (type HC3)
PPE	Personal Protective Equipment
IPC	Infection Prevention and Control
